# Raw Photoplethysmography as an Enhancement for Research-Grade Wearable Activity Monitors

**DOI:** 10.2196/57158

**Published:** 2024-09-27

**Authors:** Paul R Hibbing, Maryam Misal Khan

**Affiliations:** 1 Department of Kinesiology and Nutrition University of Illinois Chicago Chicago, IL United States; 2 Department of Kinesiology and Health Sciences University of Waterloo Waterloo, ON Canada

**Keywords:** measurement, optical sensors, sensor fusion, wearable electronic devices, accelerometry, photoplethysmography, digital health, exercise, sedentary behavior

## Abstract

Wearable monitors continue to play a critical role in scientific assessments of physical activity. Recently, research-grade monitors have begun providing raw data from photoplethysmography (PPG) alongside standard raw data from inertial sensors (accelerometers and gyroscopes). Raw PPG enables granular and transparent estimation of cardiovascular parameters such as heart rate, thus presenting a valuable alternative to standard PPG methodologies (most of which rely on consumer-grade monitors that provide only coarse output from proprietary algorithms). The implications for physical activity assessment are tremendous, since it is now feasible to monitor granular and concurrent trends in both movement and cardiovascular physiology using a single noninvasive device. However, new users must also be aware of challenges and limitations that accompany the use of raw PPG data. This viewpoint paper therefore orients new users to the opportunities and challenges of raw PPG data by presenting its mechanics, pitfalls, and availability, as well as its parallels and synergies with inertial sensors. This includes discussion of specific applications to the prediction of energy expenditure, activity type, and 24-hour movement behaviors, with an emphasis on areas in which raw PPG data may help resolve known issues with inertial sensing (eg, measurement during cycling activities). We also discuss how the impact of raw PPG data can be maximized through the use of open-source tools when developing and disseminating new methods, similar to current standards for raw accelerometer and gyroscope data. Collectively, our comments show the strong potential of raw PPG data to enhance the use of research-grade wearable activity monitors in science over the coming years.

## Introduction

Wearable monitors are increasingly used to measure physical activity in research, and new tools and techniques are continually emerging [1]. Recent innovations have improved the cost, size, and technical capability of various monitors [2], but accuracy has not increased at a commensurate pace [3-6]. Thus, there is a need for further innovation. Successful innovation will likely entail novel measurement paradigms, rather than incremental improvements on current techniques [6]. One of the most promising and underexplored paradigms is to integrate data from multiple types of sensors, rather than the traditional use of only accelerometer sensors [7,8].

**Figure 1:**
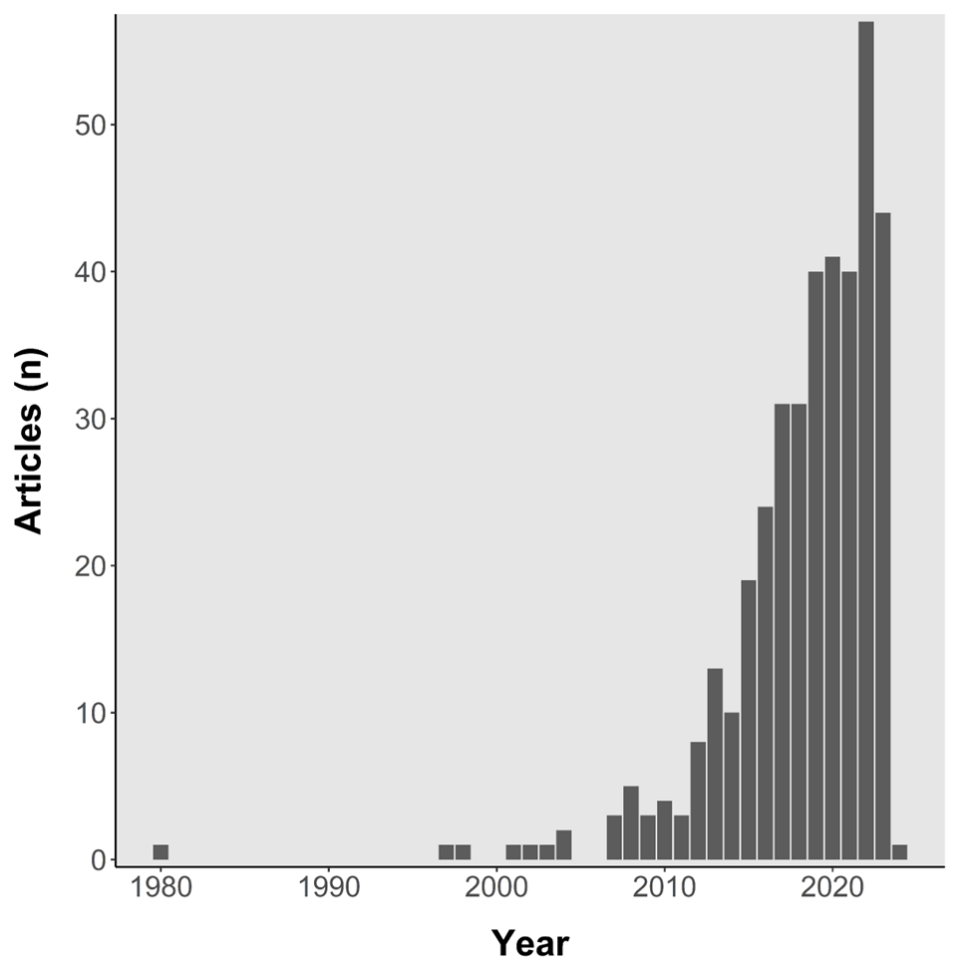
Annual publication counts over time, drawn from a Scopus.com search for “TITLE-ABS-KEY (physical AND activity AND photoplethysmography OR ppg),” conducted on December 6, 2023.

Photoplethysmography (PPG) is an optical technology that may have potential to enhance physical activity measurement when combined with established inertial sensors (accelerometers and gyroscopes) [9]. Although PPG was first described nearly 90 years ago, it has only recently gained a high level of visibility for physical activity assessment [10-12]. This growth is reflected in Figure 1, which shows the results of a Scopus search for documents addressing physical activity and PPG. Roughly half of the identified studies (183/385, 48%) were published in 2020 or later, and roughly three-quarters (285/385, 74%) were published in 2017 or later.

To date, most applications of PPG for physical activity assessment have involved consumer-grade smartwatches [13-16]. A wealth of developmental research has also been reported in the engineering literature [17-20], but commercial products for research have rarely incorporated PPG sensors and even more rarely given access to raw PPG data (ie, the data recorded by the sensor itself, without any preprocessing applied) [21]. This is beginning to change, and as it does, there is a need to raise awareness of PPG and its potential contribution to monitor-based physical activity assessment. In particular, awareness is needed for *raw* PPG data since it provides an avenue for device-agnostic measurement and iterative, open-source refinement, similar to the standard for inertial sensing [22].

In this viewpoint paper, we present raw PPG as a new frontier in monitor-based methodology. To do this, we first provide an overview of the fundamentals of PPG for physical activity assessment, after which we describe the importance and availability of raw PPG data, as well as specific applications where it holds the most potential. Throughout, we highlight ways that raw PPG data can synergize with raw data from inertial sensors to overcome long-standing challenges (eg, measurement during cycling).

## Fundamentals of PPG for Physical Activity Assessment

### Technology, Techniques, and Theory

There are 2 types of PPG, namely, transmission and reflectance [23]. Transmission PPG is common in clinical settings where it is used for pulse oximetry [12]. It typically involves red and near-infrared lights, which are shone into one side of a tissue (commonly a finger or an earlobe) and measured upon exiting the other side [10,24,25]. In physical activity assessments, transmission PPG has limited use compared with reflectance PPG. Therefore, we do not provide further comments on transmission PPG.

Reflectance PPG has been investigated using both “wearable” and “remote” instruments, the latter referring to cameras that do not touch the skin. Similar to transmission PPG, remote applications of reflectance PPG have minimal relevance for physical activity assessment, and thus we forgo additional comments on them. Instead, we focus our comments on wearable applications of reflectance PPG, particularly those embedded in wrist-worn devices. Hereafter, we use the term “PPG” to refer exclusively to such applications.

As noted by Mannheimer [26], the term “reflectance” is a misnomer, since there are no mirrors in the skin. Instead, light is scattered by various components of the tissue, and portions of the scattered light return to the surface where they can be measured by a photodetector. Thus, the defining characteristic of this PPG technique is that emission and measurement of light occur on the same side of the tissue [27].

**Figure 2:**
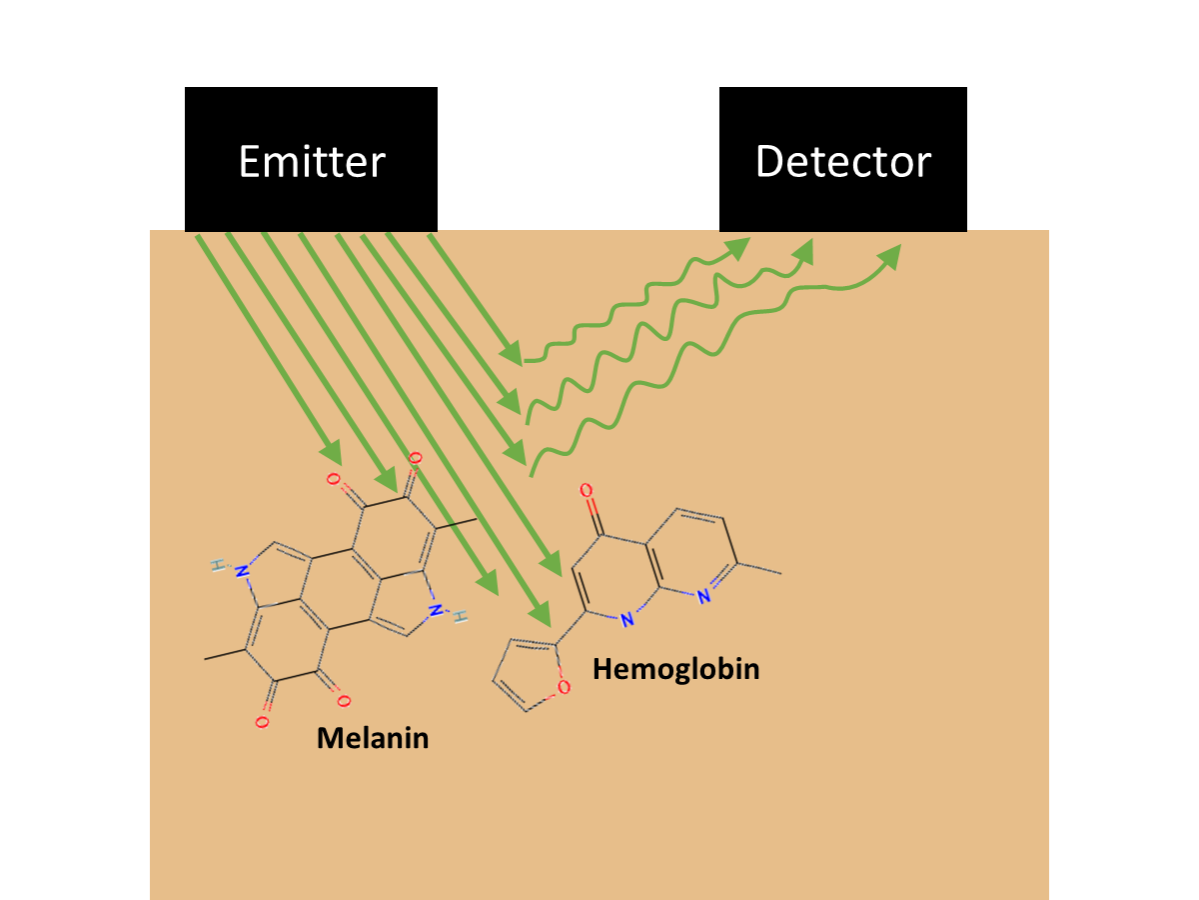
Basic representation of PPG technology. An emitter shines light into the skin. The light is absorbed by some components—mainly hemoglobin and melanin—and scattered by others toward a photodetector. Pulse waves cause increases in local blood concentration, leading the balance of absorption and scatter to shift in favor of more absorption. The photodetector signal thus diminishes as the pulse wave passes, creating a waveform that can be analyzed to predict cardiovascular parameters such as heart rate and blood pressure. Public domain icons from PubChem are shown for melanin (CID 6325610) and (deoxy)hemoglobin (CID 135310457).

There is some debate around what exactly PPG captures, but the prevailing theory is that it detects pulsatile changes in blood volume [28-30]. Figure 2 depicts the mechanics of this proposed process, with light being shone into the skin while cyclical fluctuations in scatter are monitored. These fluctuations occur because blood concentration is increased when a pulse wave passes under the light, resulting in more light absorption in accordance with the Beer-Lambert law [26,31]. Consequently, a waveform emerges in the PPG signal, which can be analyzed to detect pulse waves and calculate related parameters such as heart rate [9,10,23]. Green light is typically used because it offers shallower penetration and greater robustness against motion artifacts and other noise [17,19,32-35].

### Common Difficulties and Sources of Error

It is important to understand not only the theoretical workings of PPG but also practical issues that affect its operation. Fine et al [36] have grouped such issues into 3 categories (individual differences, physiology, and external factors), while Bent et al [37] have highlighted the unique importance of one overarching individual factor (skin type) and 2 external factors (motion artifact and signal crossover). In this section, we provide a brief overview of these latter 3 factors and their potential implications for physical activity research, with additional comments on broader sources of error for general applications of PPG.

Skin type encompasses adiposity, pigmentation, and other factors that influence tissue composition (see the study by Fine et al [36] for a detailed listing). Differences in tissue composition can affect optical scattering and absorption in ways that are difficult to predict. Accordingly, prior work has shown the accuracy of PPG-based estimates to vary depending on age, sex, obesity status, and skin tone [13,20,38-40]. The latter is an especially important variable to consider because melanin is one of the skin’s main absorbers of light at various wavelengths [41,42]. When using PPG for the measurement of physical activity, there is thus a clear need to ensure that new methods have consistent accuracy across diverse skin types. This is especially important given the implications for equity in health research.

Motion artifact is movement-induced noise in the PPG signal, which can occur due to both mechanical and physiological aspects of the movement [43]. Efforts to address motion artifact often rely on frequency-domain analyses, since it is expected that the rhythmicity of the pulse will create a sharper contrast between signal and noise in that domain [44]. Increasingly, these analyses involve cross-referencing PPG against data from concurrently worn accelerometer and gyroscope sensors to aid in differentiating between inertial and cardiovascular signal [45]. Thus, when using PPG for physical activity assessments, future studies may benefit from using devices that provide access to raw data from both PPG and inertial sensors.

Signal crossover is closely related to motion artifact and refers to confusion between rhythmic motions of the monitor itself (eg, during locomotion) and the inherent rhythmicity of the cardiovascular signal from PPG [37]. Specifically, cardiovascular signal is expected to have dominant frequencies between roughly 1.0 Hz and 3.5 Hz (corresponding to heart rates between 60 and 210 beats per minute), and human movements can generate considerable amplitude in the same range [46-48]. Thus, it is likely that some motions will result in overlap of inertial and pulsatile signal components, making it hard to tell which is which. This is one reason that PPG-based devices have frequently been shown to have lower accuracy during physical activity than during other behaviors [37,49,50]. Signal crossover is uniquely important to highlight because it suggests that device accuracy may vary based on not only the amount of movement but also the type of movement. This could have major implications for physical activity assessments, making it crucial to address in future work.

Apart from skin type, motion artifact, and signal crossover, Fine et al [36] have highlighted difficulties posed by physiological factors (respiration, venous pulsations, attachment site of the device, and body temperature) and additional external factors (ambient light and pressure of the sensor on the skin). These difficulties are important to acknowledge and address, but their implications may not be substantively different for physical activity research than what has been described for other disciplines.

Many of the difficulties with raw PPG resemble what is already faced when dealing with inertial data from accelerometers and gyroscopes [51]. The latter sensors have enhanced the measurement of physical activity despite their limitations [52], and thus PPG may have similar potential. Furthermore, the impact of device limitations may diminish over time through ongoing innovation in technology and analytics. Thus, the difficult aspects of PPG can be viewed as opportunities for refinement rather than insurmountable barriers.

## Importance and Availability of Raw PPG Data

To understand the revolutionary potential of raw PPG data for physical activity assessment, it is helpful to consider a similar revolution that has already taken place with accelerometer data [53-55]. Historically, accelerometer-based devices provided only proprietary “activity counts” as their output, which led to intermonitor differences and a lack of flexibility to innovate with new data processing techniques [56-58]. Over time, raw acceleration data became commonplace, opening doors for standardization and innovation in physical activity research [2]. An especially noticeable result was that many researchers began to focus on techniques that combined research-grade products with open-source tools for data processing and analysis, thereby promoting streamlined and coordinated progress in the field [59-64].

The potential parallels for PPG data are striking. To date, most research with PPG has relied on proprietary outputs from consumer-grade devices, which have been used to track heart rate, atrial fibrillation, blood pressure, and more [65-76]. Intermonitor differences and lack of flexibility are thus limitations of current standards for PPG, in much the same way as they once were for accelerometry. Furthermore, concerns have frequently been raised about unannounced algorithm and firmware updates that can make consumer-grade technology undesirable in certain research contexts [77-81]. The advent of raw PPG data therefore offers many of the same benefits that have already been derived from raw accelerometer data, especially when pairing research-grade devices with open-source resources. But the full potential of raw PPG for physical activity research cannot be realized unless the market provides devices that are scalable for use in large studies.

Existing research involving raw PPG data has generally involved small-scale devices (sometimes custom-made) [19,21,27,30,82], specialized tools for hospital use [83-86], or smartphone technology [87-89]. While these studies have shown strong proof-of-concept, they have only sometimes been oriented toward physical activity research, and the availability of suitable devices for large assessments remains an issue. The best-known research-grade devices are likely the E4 and EmbracePlus from Empatica Inc, the Shimmer3+ GSR from Shimmer Sensing, and the LEAP from ActiGraph LLC. Each device has strengths and limitations, with major points of comparison being cost, comfort, and access to raw data. The Shimmer3+ GSR is the most affordable option and provides access to fully raw data from both PPG and inertial sensors. However, a potential limitation is its reliance on physical components (eg, wired probes that wrap around the fingers) that may be unappealing or uncomfortable for some participants. The EmbracePlus is the most expensive device and is a replacement for the E4. It is designed like a standard smartwatch and is therefore very comfortable, but it does not provide truly raw PPG data (nor did the E4 [21]). Specifically, the EmbracePlus preprocesses PPG data using a proprietary algorithm that produces a blood volume pulse waveform, which resembles but does not replace raw data as it would appear in a direct recording from the photodetector. The LEAP device falls between the other 2 in terms of cost and comfort but does provide access to fully raw data from PPG and inertial sensors.

As new and upgraded devices continue to emerge and provide access to raw PPG data, a key objective will be to apply, extend, and standardize the techniques from earlier proof-of-concept studies for use in large-scale physical activity assessments for research. The following section outlines several specific areas in which there may be greatest warrant for these efforts.

## Potential Applications of Raw PPG in Assessments of Physical Activity

The most obvious application of raw PPG for physical activity assessment is heart rate monitoring, where a notable contrast exists between the wrist-based optical approach and standard electrode-based approaches involving chest-worn monitors (eg, heart rate straps and Holter monitors). The latter tend to have greater accuracy than the former [90,91] and yet can also be uncomfortable to wear, especially over long periods [92]. Conversely, wrist-based PPG devices can be comfortably worn over long periods and yet have lower accuracy than chest-worn monitors. One implication is that raw PPG may encourage participant compliance in long assessment protocols (eg, lasting a week or more, which is common in physical activity research). This could be especially valuable for interventions that assess change over time, since responsiveness is generally a greater concern than accuracy in those contexts. Moreover, the diminished accuracy compared with chest-worn monitors may be less of an issue in cases where the key outcome is categorical intensity rather than continuous heart rate (eg, if assessing time in heart rate zones, where measurement error would be a concern only at the boundaries between zones, rather than across the spectrum of continuous heart rates). Nevertheless, there is a definite trade-off between accuracy and comfort, and neither chest-worn nor wrist-worn monitors are the optimal choice for every research question. This makes the accuracy-comfort trade-off a critical consideration when selecting a monitor for research. With continued innovation and refinement, the accuracy gap may narrow between chest- and wrist-worn devices, and trade-off–related considerations may change accordingly. But it is unlikely that the issue will ever disappear completely.

While heart rate monitoring is an obvious application for raw PPG, it may not be the most impactful one. Rather, there may be greater promise when combining raw data from PPG and inertial sensors to predict other physical activity–related outcomes such as energy expenditure. This multimodal approach not only allows for robust correction of motion artifact in the PPG signal (as described previously) but also enables concurrent analysis of movement and cardiovascular data. While this is not an entirely new concept, the ability to carry it out using purely raw data from a single wrist-worn device is quite recent. Thus far, the primary multimodal methods have relied on either separately worn movement and cardiovascular monitors [93-95] or the chest-worn Actiheart device (CamNtech Ltd) [96-99] when predicting energy expenditure. These approaches have shown clear synergy between movement and cardiovascular data but have ultimately had limited uptake compared with the widespread use of wrist-worn monitors in field-based research. Furthermore, heart rate has been the only cardiovascular parameter emphasized with the earlier methods, whereas raw PPG can potentially lead to enhanced predictions through the capture of additional aspects of cardiovascular response to activity (eg, pulse wave parameters and variability). This highlights the warrant for translating and extending earlier concepts of multimodal assessment to the use of raw PPG and inertial data from wrist-worn devices.

The combination of raw PPG and inertial data may also help overcome known limitations of movement-only techniques in the prediction of energy expenditure. For example, wrist-worn monitors are generally unable to register any motion during cycling despite the level of lower-limb exertion, resulting in poor measurement validity [100]. In contrast, PPG may still detect exertion during cycling because it relies on optical and physiological signal rather than inertial signal. This advantage reflects the known benefit of using not only multiple sensors but multiple types of sensors [51,56]. Similar benefits may also arise for other activities where the body’s inertial profile is altered, such as when carrying an external load or pushing a stroller [101,102]. The potential to overcome these limitations with virtually no change in participant burden highlights the strong potential of raw PPG to improve physical activity research, especially in the area of energy expenditure prediction.

Combining raw PPG and inertial data may also be beneficial for activity recognition, promoting greater understanding about certain elements of activity context [103]. Activity recognition is also important as a precursor to energy expenditure prediction, since it is much easier to predict energy expenditure if the type of activity is first known [104-106]. This is the basis for several prior models of energy expenditure, including well-known 2-regression models [107-114]. The utility of raw PPG for activity recognition was recently highlighted by Hnoohom et al [115], who calibrated models using data from 3 open-access datasets [116-118]. The parent studies used devices from Shimmer, Empatica, and Maxim Integrated (Analog Devices Inc), and the models were calibrated using deep learning and different combinations of accelerometer, PPG, and electrocardiographic data. When combining accelerometer and PPG data, the dataset-specific models each achieved near-perfect accuracy during 10-fold cross-validation. However, external validations are needed to confirm the effectiveness of the models and apply them with scalable devices, as described previously.

Outside of energy expenditure and activity recognition, raw PPG may have use for measuring a range of other physical activity–related variables as well, from specific hemodynamic parameters related to exertion (eg, pulse transit time [119]) to consequences of physical activity such as fatigue and recovery [120]. Steps warrant mention as well, given their status as a well-known output of most wearable activity monitors. Raw PPG could potentially enhance or refine the measurement of steps and related variables (eg, cadence), including during periods where the inertial movement profile is altered, as described previously for energy expenditure. The combination of raw data from PPG and inertial sensors could also enable automated measurement of highly specialized outcomes in free-living settings, such as cardiac-locomotor coupling (ie, synchrony of footfalls with systole or diastole) [121].

Finally, although our focus has been on physical activity–related applications of raw PPG, it is important to acknowledge potential contributions in the broader context of 24-hour measurement as well. This refers to the growing emphasis on interrelationships between physical activity, sedentary behavior, and sleep as part of a daily composite [122,123]. The importance of the concept is reflected in the release of 24-hour movement guidelines from numerous governments and the World Health Organization over the last few years [124-129], and there are at least 2 key contributions raw PPG can make to 24-hour assessments. One is to differentiate between nonwear, sedentary behavior, and sleep, all of which produce minimal accelerometer and gyroscope signal and are therefore hard to tell apart using only inertial data. Raw PPG may exhibit richer variation across the categories, thereby assisting with disambiguation. Some proof-of-concept already exists in this area as well, given the amount of prior work using PPG for sleep measurement [130]. The other benefit may be to assist with classifying posture (seated or lying vs upright) [131], which is essential for differentiating sedentary behavior from light-intensity physical activity [132]. These possibilities highlight the strong potential and exceptional flexibility of raw PPG, which will be an asset for a broad range of movement-oriented research in the coming years.

## Discussion and Conclusion

In this viewpoint paper, we have introduced raw PPG and highlighted its potential benefits for physical activity assessments. Our specific focus on raw PPG data (as opposed to preprocessed or aggregated data) was critical and timely, given the recent emergence of mainstream devices that provide access to them. A key strength of raw PPG is that its optical basis complements the inertial basis of familiar accelerometer and gyroscope sensors. Furthermore, raw PPG can be used to assess not only heart rate but also broader aspects of cardiovascular physiology. These are the driving forces behind the potential we laid out in the prior sections.

Going forward, it will be critical to obtain raw data from both PPG and inertial sensors, not only to facilitate merging them but also to make new algorithms both transparent and device agnostic (ie, applicable to data from any PPG-inclusive device). These characteristics help combat the “black box” phenomenon of closed-source devices [133]. Device agnosticism also plays an important role in “future proofing” new methods by reducing dependence on individual monitors that can leave the market at any time (eg, as seen with the SenseWear Armband, Phillips Actiwatch, Empatica E4, and ActiGraph GT9X). The use of raw PPG can be further advanced by using open-source channels when developing and disseminating of new resources, consistent with growing standards for existing wearable devices in physical activity assessment [59-64].

This viewpoint paper was among the first to suggest the value of integrating raw PPG data into large-scale assessments of physical activity, where the importance of raw accelerometer and gyroscope data has already been established. In doing so, the viewpoint paper serves to orient new users to the wealth of prior work on PPG from other research areas, where critical reference points have been provided that can spur a paradigm shift in physical activity research. A noteworthy limitation of the viewpoint paper was that it was not a systematic review. As such, it did not fully summarize the available literature, whether in general or focused specifically on physical activity assessment. Nevertheless, our overall conclusion is that there is warrant for vigorous exploration of raw PPG going forward.

## Abbreviations

PPG: photoplethysmography
